# Co-Targeting of JNK and HUNK in Resistant HER2-Positive Breast Cancer

**DOI:** 10.1371/journal.pone.0153025

**Published:** 2016-04-05

**Authors:** Kendall Phelps-Polirer, Melissa A. Abt, Danzell Smith, Elizabeth S. Yeh

**Affiliations:** 1 Department of Health Sciences, Clemson University, Columbia, SC, United States of America; 2 Department of Cell and Molecular Pharmacology and Experimental Therapeutics, Medical University of South Carolina, Charleston, SC, United States of America; 3 Department of Pathology and Laboratory Medicine, Medical University of South Carolina, Charleston, SC, United States of America; University of South Alabama Mitchell Cancer Institute, UNITED STATES

## Abstract

Strategies for successful primary treatment of HER2-positive breast cancer include use of the HER2 inhibitors trastuzumab or lapatinib in combination with standard chemotherapy. While successful, many patients develop resistance to these HER2 inhibitors indicating an unmet need. Consequently, current research efforts are geared toward understanding mechanisms of resistance and the signaling modalities that regulate these mechanisms. We have undertaken a study to examine whether signaling molecules downstream of epidermal growth factor receptor, which often act as compensatory signaling outlets to circumvent HER2 inhibition, can be co-targeted to overcome resistance. We identified JNK signaling as a potential area of intervention and now show that inhibiting JNK using the pan-JNK inhibitor, SP600125, is effective in the HER2-positive, resistant JIMT-1 xenograft mammary tumor model. We also investigate potential combination strategies to bolster the effects of JNK inhibition and find that co-targeting of JNK and the protein kinase HUNK can prohibit tumor growth of resistant HER2-positive mammary tumors *in vivo*.

## Introduction

~15–25% of breast cancer patients overexpress the human epidermal growth factor receptor (EGFR) family member HER2. While targeted therapies including trastuzumab and lapatinib are available to treat HER2-positive (HER2+) breast cancer, many patients become resistant to these inhibitors. Consequently, much research effort is geared toward understanding mechanisms of resistance including upregulation and activation of compensatory signaling pathways such as additional growth factor receptors or molecules that circumvent HER2 inhibition by inducing downstream effector pathways. Experimental testing and clinical application of combination therapy provides significant opportunity to overcome therapeutic resistance in breast cancer [1–3].

Although trastuzumab and lapatinib both inhibit HER2, their mechanisms of action differ. Trastuzumab is a humanized monoclonal antibody that targets one of the extracellular domains of HER2 [4]. It has been reported to induce HER2 internalization in a c-Cbl, E3 ligase, dependent fashion [5]. Conversely, lapatinib is a potent yet reversible small molecule that acts on the intracellular ATP binding site in the kinase domain of HER2. Lapatinib is also active against EGFR. When resistance develops, similar and differing mechanisms have been reported for trastuzumab and lapatinib. Upon developing resistance to trastuzumab, reliance on other HER-family members, including EGFR, or overexpression of ligands for EGFR family members are common mechanisms that have been described [6–10]. Thus, application of lapatinib, which also targets EGFR often remains beneficial. Activation of Akt signaling, either through loss of PTEN or activation of PI3K is implicated in both intrinsic and acquired resistance to HER2 inhibitors [6, 11–14]. Activation of Akt is able to compensate for loss of HER2 signaling and therefore, increased levels of Akt activity have also been shown to predict response to HER2 inhibition, where high levels of Akt corresponds to poorer response to HER2 inhibitors [11]. Therefore, evaluating signaling molecules downstream of HER2 and EGFR signaling may provide clues to which compensatory signaling molecules are most robustly activated during the acquisition of resistance.

Using BT474 cells as the primary inhibitor sensitive cell line and JIMT-1 as the resistant cell model, we were able to confirm Akt as a major factor in both the primary and resistant cell lines. More importantly, we also found that an inhibitor that targets the protein kinase JNK was effective, which we now report as a potential target using the JIMT-1 HER2-resistant model [15], as well as a BT474-based lapatinib resistant model (BT474-LR). Consequently, we initiated investigation on the effect of targeting JNK in these models. Our findings show that JNK inhibition using the pan-JNK inhibitor SP600125 was effective in inducing cell death in JIMT-1 and BT474-LR resistant cell lines as well as impairing tumor growth of JIMT-1 mammary tumor xenografts. We went on to test the JNK inhibitor as part of a combination regimen. We found that JNK targeting effectively combined with targeting of the HUNK kinase, which was also recently described as a potential target using the JIMT-1 model [16]. Taken together, our findings identify JNK as a potential target using the JIMT-1 and a BT474-LR HER2-inhibitor resistant breast cancer models and we put forward a potential combination strategy that applies co-targeting of JNK and HUNK kinases.

## Materials and Methods

### Cell Culture

All cells were maintained at 37°C and 5% CO_2_. BT474 cells were grown in RPMI-1640 (Corning) supplemented with 10% fetal bovine serum (FBS, Gibco). JIMT-1 (Addex Bio) cells were grown in DMEM (Corning) supplemented with 10% FBS. JIMT-1 cells expressing control or HUNK shRNA were generated as previously described [16]. All media contained 2 mM glutamine (Corning) and Penicillin/Streptomycin (Pen/Strep, Corning) unless otherwise specified. Inhibitors for AKT (AKt Inhibitor VIII), PLCγ (Et-18-OCH_3_), JNK (SP600125), PI3K (LY294002), SRC (PP2), mTORC1 (Rapamycin), p38 (SB 203580), JAK (AG 490), and c-RAF (ZM 336372) were purchased from EMD Millipore. Lapatinib was purchased from Santa Cruz.

### Immunoblotting

Cells were lysed in buffer containing final concentrations of 50 mM Tris-HCl, pH 7.5; 150 mM NaCl; 1% Triton X-100; 0.1% SDS supplemented with HALT protease and phosphatase inhibitor cocktail (Thermo Scientific). Primary antibodies used for western blotting are: anti-LC3B (Cell Signaling-2775), anti-p-JNK (Cell Signaling-4668), JNK (Cell Signaling-9258), p-AKT (Cell Signaling-4060), AKT (Cell Signaling-4691), p-EGFR (Cell Signaling-3777), EGFR (Santa Cruz-sc-373746) and anti-β-tubulin (Santa Cruz-sc-55529). Imaging and quantitation was performed on the Odyssey imaging system (LICOR) or the FluorChem-R (ProteinSimple). Quantitation was normalized to β-tubulin as a loading control before determining the fold change in protein levels.

### RNA isolation and quantitative RealTime PCR

RNA was prepared by using the GeneJet RNA isolation kit (Thermo Scientific). Reverse transcription was performed using the Maxima First Strand cDNA Synthesis Kit for RT-PCR (Thermo Scientific). The resulting cDNA was used to perform quantitative RealTime PCR (QRT-PCR) using the Bio-Rad myIQ system. PrimePCR SYBR Green Assay for human HUNK was purchased from Bio-Rad. Primers for GAPDH are Forward-TGCACCACCAACTGCTTAGC and Reverse-GGCATGGACTGTGGTCATGAG.

### Crystal Violet Stain Analysis

Equal numbers of cells (5000 cells/well) were plated and treated the following day with 1 μM lapatinib for 96 hr. Following drug treatments, cells were fixed in 4% paraformaldehyde and stained with crystal violet for 1hr. Cells were subsequently washed in deionized H_2_O, allowed to dry, and crystal violet was extracted with methanol. A540 was read using Benchmark Plus plate reader (Biorad).

### Caspase-3 Activity

Equal numbers of cells (5,000 or 20,000 cells/well) were plated on 96-well dishes and treated the following day with indicated inhibitors for 24 hours prior to analysis by Caspase-3 activity assay (Sigma) as previously described [16].

### Animal Care

Animal care and all animal experiments were performed with the approval and in accordance with the guidelines of the Medical University of South Carolina IACUC. All mice were housed and cared for in the Animal Research Center at Medical University of South Carolina, which is AAALAC accredited facility. Mice were housed in a BSL2 rooming facility for immunocompromised animals in individually ventilated racks with sterile water, and their own food. Animals were euthanized by anesthesia overdose with isofluorane in accordance with the *Guide for the Care and Use of Laboratory Animals*. Protocols were in place for early and humane endpoints in the event that an experimental animal displayed signs of illness, such as poor body condition, lethargy, piloerection, and lack of grooming behavior, prior to the experimental endpoint. To determine when/if animals should be euthanized, tumor measurements and health monitoring of experimental animals was performed regularly by lab and veterinary staff. For the experiments currently represented in this study no animals died prior to the experimental endpoint.

### In vivo tumorigenesis

For orthotopic tumor analysis, 5 x 10^6^ cells were injected in the abdominal mammary fat pat of immunocompromised mice (Nu/J-Foxn1^nu/nu^, Jackson Labs). Tumors were evaluated by manual palpation using calipers. When tumors reached a ~volume of 60mm^3^, drug treatments were initiated. For *in vivo* drug treatments chloroquine (Sigma) solubilized in sterile saline was delivered by i.p. injection at 50 mg/kg. Lapatinib (LC Laboratories) was resuspended in a solution containing 0.5% hydroxypropylmethylcellulose; 0.1% Tween-20 and delivered orally at 100 mg/kg. SP600125 (Selleck) was resuspended in a solution containing 30% PEG-400; 5% polypropylene glycol; 0.5% Tween-80 and delivered by i.p. injection at 30 mg/kg. Placebo for each experiment is the vehicle in which each drug was reconstituted.

### Statistical Analysis

As indicated in the figure legends, p-values for *in vitro* experiments were analyzed using Student’s T-test. For tumor studies, Kaplan-Meier survival curves were used to estimate group-specific median time to tumor volume of 600 mm^3^ and p values were analyzed by Wilcoxon signed-rank test (log-rank test).

## Results

### JNK is a target in HER2 inhibitor resistant human breast cancer cells

**Fig 1:**
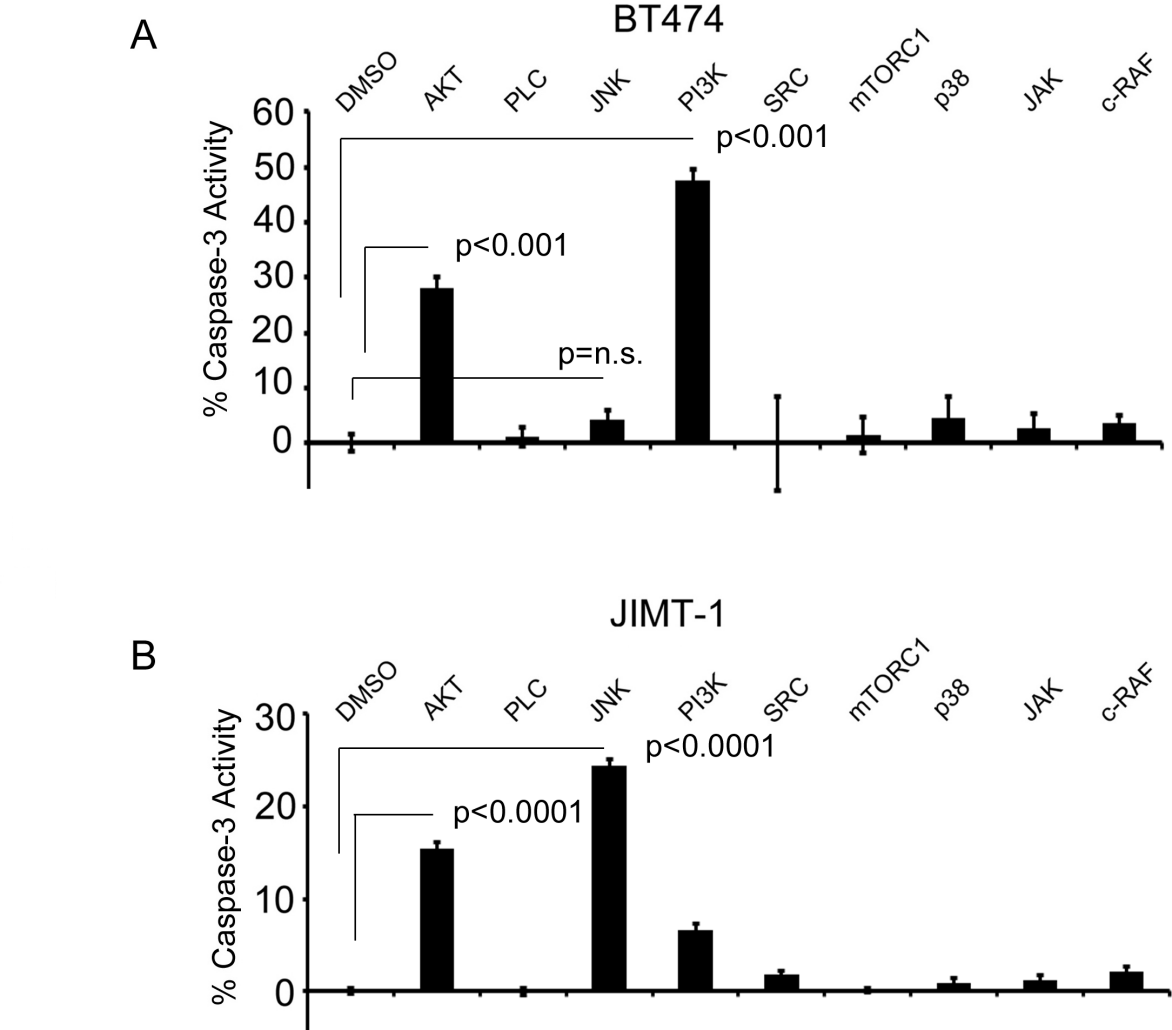
JNK inhibition with SP600125 in JIMT-1 cells induces cell death. A) BT474 cells or B) JIMT-1 cells treated with inhibitors targeting AKT, PLC, JNK, PI3K, SRC, mTORC1, p38, JAK, and c-RAF for 24 hrs and evaluated for Caspase-3 activity as a measure of apoptosis. p-values were determined by student’s T-test. n.s. = not significant

To determine the importance of specific signaling molecules in HER2+ breast cancer cells that are sensitive to HER2 inhibitors or have been reported to be resistant, we evaluated a panel of inhibitors toward AKT, PLCγ, JNK, PI3K, SRC, mTORC1, p38, JAK, and c-RAF on BT474 (sensitive) and JIMT-1 (resistant) human breast cancer cells in an *in vitro* (2D culture) cell death analysis. In line with previous observations, it was clear that the PI3K-AKT pathway played a significant role in both inhibitor sensitive and insensitive HER2+ cell lines. In BT474 cells AKT inhibition and PI3K inhibition significantly induced Caspase-3 activity compared to BT474 cells treated with DMSO vehicle alone (Fig 1A). Similarly, AKT inhibition also induced Caspase-3 activity in JIMT-1 cells compared to JIMT-1 cells treated with DMSO vehicle alone (Fig 1B). However, these cells were only moderately responsive to PI3K inhibition when compared to levels of responsiveness to Akt inhibition (~6% versus ~15% respectively), which is consistent with reports that AKT is active in JIMT-1 cells in a manner that circumvents HER2 activation and possibly uncouples from PI3K [12, 15]. We did not observe any significant induction of cell death in response to PLCγ, SRC, mTORC1, p38, JAK, or c-RAF inhibitors in either cell line.

Most significantly, we found that the JIMT-1 cells responded to JNK inhibition when compared to the JIMT-1 cells treated with DMSO (Fig 1B), which we did not robustly observe in the BT474 cell line as the Caspase-3 activity induced in BT474 cells treated with JNK inhibitor was not statistically significant when compared to BT474 cells treated with DMSO (Fig 1A), suggesting that JNK signaling could play a role in regulating the survival of HER2+ breast cancer cells that are resistant to HER2 inhibitors. We also generated a lapatinib resistant cell line by culturing BT474 cells continuously in lapatinib up to 1 μM concentration (BT474-LR) and assayed these cells for lapatinib resistance using a chrystal violet viability assay, which showed that the BT474-LR cells survived 1 μM lapatinib treatment whereas control cells do not (S3A Fig). When we evaluated these cells for sensitivity to AKT and JNK inhibition, we found that they responded to these inhibitors by inducing Caspase-3 activity, similar to the JIMT-1 cell line (S3B Fig), confirming our findings.

### JNK inhibition in vivo impairs tumor growth

**Fig 2:**
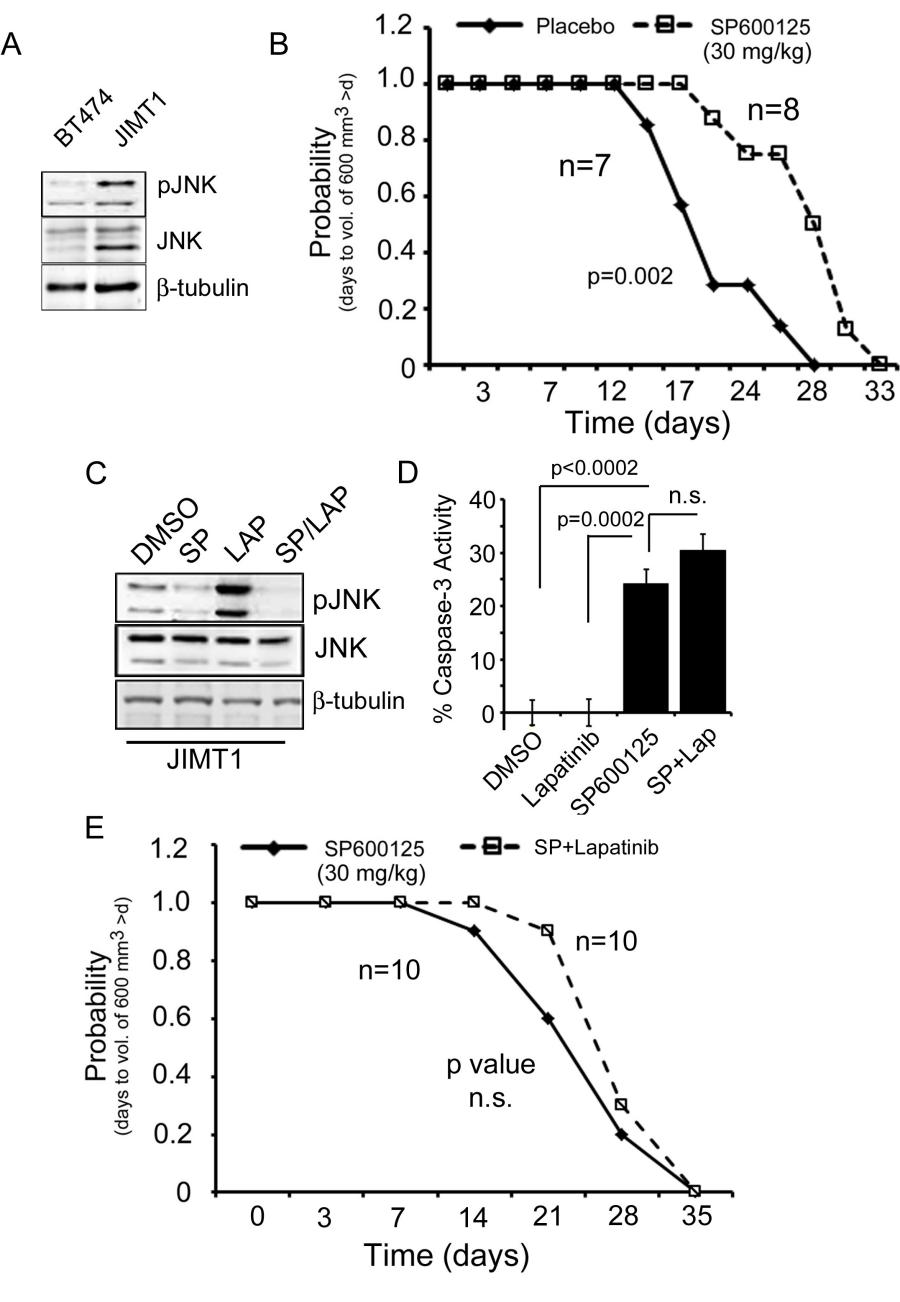
SP600125 inhibits mammary tumor growth of JIMT-1 xenograft tumors but combined lapatinib and JNK inhibition provides no added benefit. A) Immunoblot analysis of BT474 and JIMT-1 cell lysates for phospho-JNK and total JNK expression. B) Time to tumor size of 600 mm^3^ was significantly longer for SP600125 treated mice relative to placebo treated mice based on Kaplan–meier survival curve estimates; p = 0.002, log-rank test. n = 7, placebo. n = 8, SP600125. C) Immunoblot analysis of JIMT-1 cell lysates for phospho-JNK and total JNK after treatment of cells with DMSO, SP600125 (50 μM), lapatinib (1 μM), or a combination of SP600125 and lapatinib. D) JIMT-1 cells were treated with DMSO, SP600125 (50 μM), lapatinib (1 μM), or a combination of SP600125 and lapatinib for 24 hrs and evaluated for Caspase-3 activity as a measure of apoptosis. E) Time to tumor size of 600 mm^3^ was not different for mice treated with SP600125 (30 mg/kg), relative to mice treated with lapatinib (100 mg/kg) and SP600125 based on Kaplan–meier survival curve estimates. n = 10 animals per treatment group. p-value was not significant (n.s.) by log rank test.

To further investigate the role of JNK in resistance, we next probed the BT474 and JIMT-1 cells for levels of total and phosphorylated JNK and found higher expression of total JNK in the JIMT-1 cell line as compared to the BT474 cell line, with a concomitant increase in phosphorylation of JNK in the JIMT-1 cells (Fig 2A). Given our *in vitro* findings that JNK is upregulated in JIMT-1 cells compared to BT474 cells and that these cells are sensitive to JNK inhibition, we questioned whether targeting JNK *in vivo* would impair tumor growth. When we evaluated JIMT-1 derived xenograft tumor growth in mice treated with either placebo, the vehicle in which the inhibitor was mixed (see Methods section), or the JNK inhibitor, SP600125 (30 mg/kg), we found a significant delay (6.9 days) in the median time for the tumors of SP600125 treated animals to reach a volume of ~600 mm^3^ (Fig 2B).

### Combined JNK inhibition and lapatinib treatment in vivo

It was previously reported that the JIMT-1 model is resistant to lapatinib [15]. Therefore, we sought to further investigate whether SP600125 could be used in combination with lapatinib to boost the therapeutic effect of the inhibitor in this preclinical breast cancer model. First, we evaluated how lapatinib affected JNK signaling. JIMT-1 cells were treated with vehicle (DMSO), SP600125, lapatinib, or a combination of SP600125 and lapatinib then probed for phospho-JNK to assess JNK activity. We found lapatinib treatment strongly induced JNK phosphorylation, which could point to JNK having a pro-tumorigenic role in the JIMT-1 model. However, combined treatment with SP600125 and lapatinib blocked the upregulation of this phosphorylation event (Fig 2C). Therefore, we next evaluated the effect of combined JNK inhibition and lapatinib treatment on cell death and tumor growth respectively. Despite the blockade of JNK activation by the combined use of SP600125 and lapatinib, we did not observe an increased benefit of using both inhibitors either *in vitro* by Caspase-3 activity assay (Fig 2D) or *in vivo* by tumor analysis (Fig 2E).

### JNK inhibition upregulates autophagy

**Fig 3:**
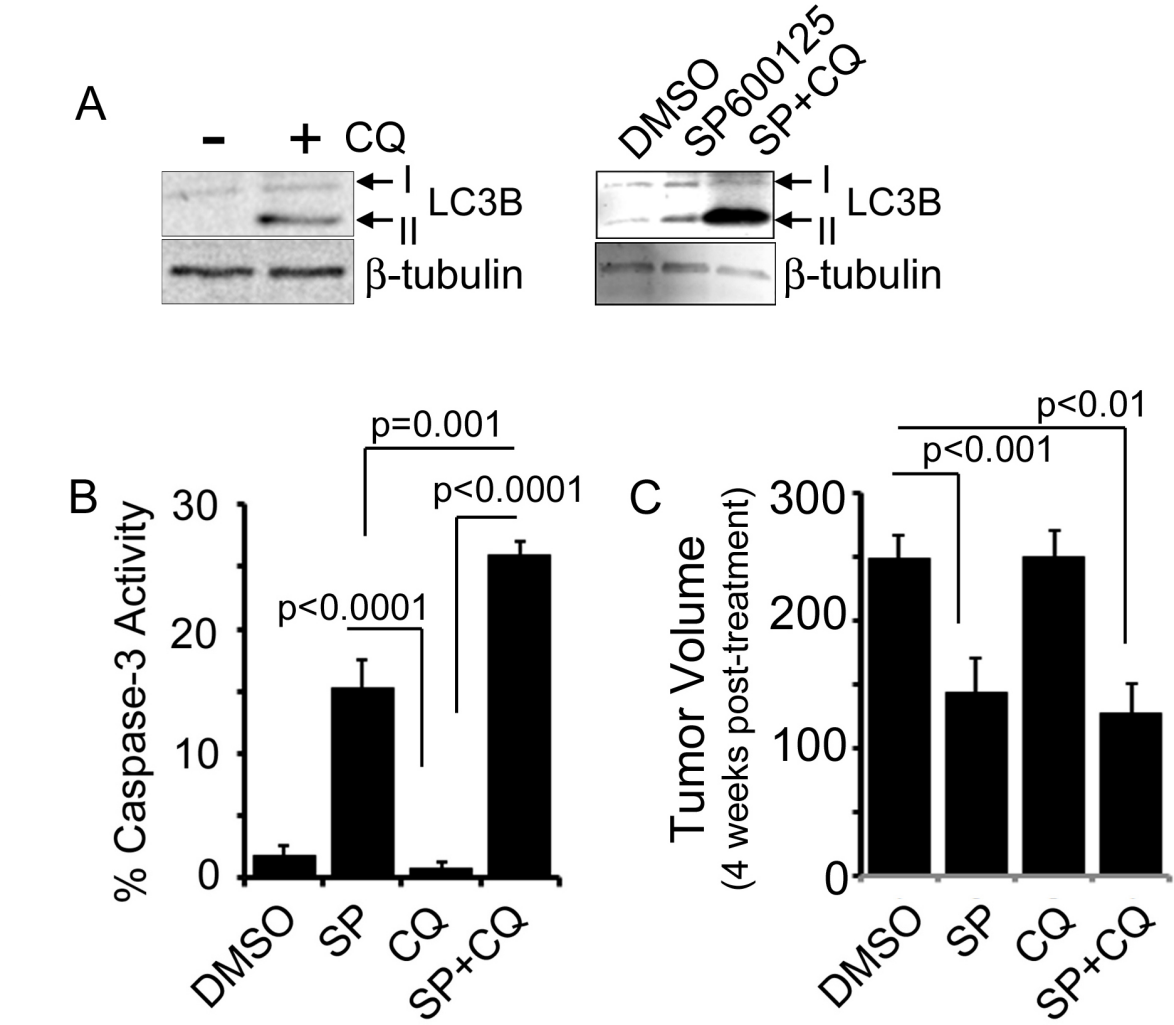
SP600125 induces autophagy *in vitro* but choroquine does not have an additive effect on inhibiting mammary tumor growth when used with JNK inhibitor *in vivo*. A) Immunoblot analysis of JIMT-1 cell lysates for LC3B I and II after treatment of cells +/- chloroquine (left panel) as well as with DMSO, SP600125 (50 μM), or SP600125 and chloroquine (100 ng/ μl). (right panel) B) JIMT-1 cells were treated with DMSO, SP600125 (50 μM), chloroquine (100 ng/ μl), or a combination of SP600125 and chloroquine for 24 hrs and evaluated for Caspase-3 activity as a measure of apoptosis. C) Average tumor volume at day 14 after treatment of placebo treated versus chloroquine (50 mg/kg) treated animals harboring JIMT-1-derived xenograft mammary tumors. n = 10 animals per treatment group. p-values were determined by student’s T-test.

While our findings suggest that JNK plays an active role in promoting survival of resistant HER2+ breast cancer cells, our findings in Fig 2 suggest that JNK is not able to act in conjunction with lapatinib to further impair tumorigenesis. Therefore, we next sought to evaluate other mechanisms of combined inhibition that could be exploited in conjunction with JNK inhibition to further improve therapeutic outcome. Several previous studies implicate activation of autophagy as a mechanism for acquiring resistance using the JIMT-1 model [16–18]. To investigate whether using the SP600125 JNK inhibitor induces autophagy we treated cells with the drug on its own or in conjunction with the autophagy inhibitor, chloroquine, to account for autophagic flux (Fig 3A-right panel). Cells were also treated with chloroquine alone to demonstrate that inhibiting autophagic flux with chloroquine induces LC3II formation as expected (Fig 3A-left panel). We found that SP600125 treatment alone induced LC3B lipidation from LC3B I to LC3B II, similarly to CQ treatment alone, suggesting that autophagy may be increased due to JNK inhibition. We further found that when JIMT-1 cells were treated with SP600125 in the presence of chloroquine, LC3B II levels were increased even more, confirming that SP600125 induces autophagy when autophagic flux is blocked by chloroquine (Fig 3A).

Although cholorquine is used in our *in vitro* experiment to demonstrate an increase in autophagy, it is pharmacologically an autophagy inhibitor that acts by preventing acidification of lysosomes, thereby blocking late stages of autophagy induced degradation of autophagosome contents. Consequently, it is used as a chemical inhibitor of autophagy *in vivo*. Therefore, to test whether inhibiting autophagy with chloroquine might enhance SP600125’s ability to promote cell death in JIMT-1 cells we evaluated Caspase-3 activity after SP600125 or chloroquine treatment alone as well as combined SP600125 and chloroquine treatment. We found that chloroquine treatment alone had no effect but that combined JNK inhibition and chloroquine increased the cell death response more robustly than SP600125 treatment alone (Fig 3B). Following we evaluated a combined SP600125 and chloroquine treatment *in vivo* but were surprised to find that unlike our *in vitro* cell death assay, we found no benefit on *in vivo* tumorigenesis with this combination (Fig 3C).

### Combined JNK inhibition and HUNK targeting

**Fig 4:**
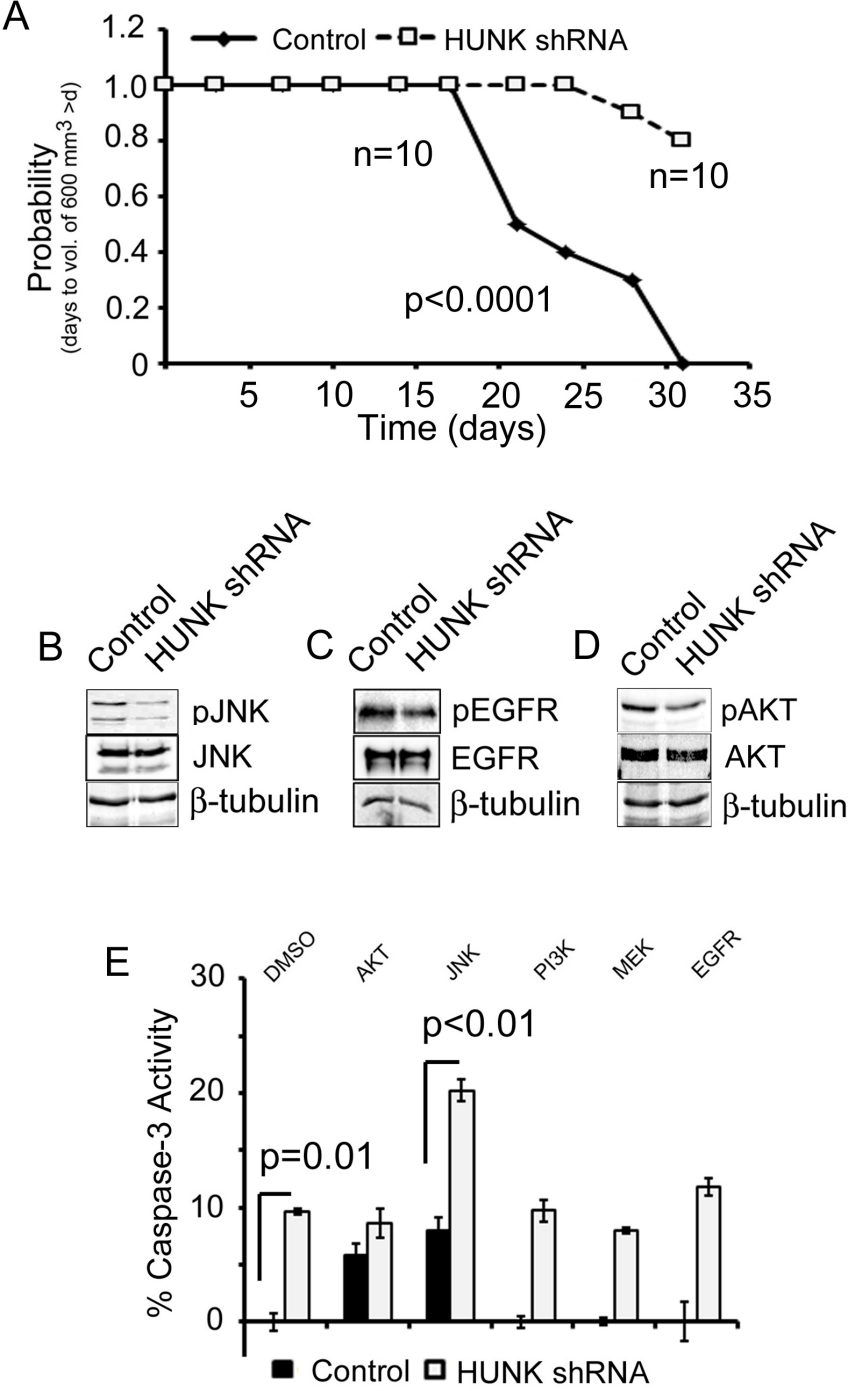
HUNK knockdown inhibits JIMT-1 xenograft mammary tumor growth. A) Time to tumor size of 600 mm^3^ was significantly longer for mice with HUNKshRNA expressing tumors relative to control shRNA derived tumors based on Kaplan–meier survival curve estimates; p < 0.0001, log-rank test. B) Immunoblot analysis of control and HUNK shRNA expressing JIMT-1 cell lysates for phospho-JNK and total JNK expression. C) Immunoblot analysis of control and HUNK shRNA expressing JIMT-1 cell lysates for phospho-EGFR and total EGFR expression. D) Immunoblot analysis of control and HUNK shRNA expressing JIMT-1 cell lysates for phospho-AKT and total AKT expression E) Control and HUNK shRNA expressing JIMT-1 cells were treated with inhibitors targeting AKT, PLC, JNK, PI3K, MEK, and EGFR for 24 hrs and evaluated for Caspase-3 activity as a measure of apoptosis. p-values were determined by student’s T-test.

While chloroquine is a commonly used autophagy inhibitor, its activity is restricted to later stages of autophagy when the lysosome is active. To explore other methods of targeting autophagy we next turned to evaluate a protein kinase called HUNK, which we recently described as an activator of autophagy in HER2+ breast cancer cells, potentially contributing to trastuzumab and lapatinib resistance [16]. HUNK has also been previously described as a downstream effector of HER2 that becomes upregulated in response to HER2 oncogene activation [19]. Therefore, we evaluated targeting of HUNK in the JIMT-1 model. Consistent with our previous findings, HUNK knockdown by shRNA (S1A Fig), as compared to JIMT-1 cells expressing control shRNA, impaired tumor growth by prompting a significant delay (18.4 days) in the median time for the tumors of HUNK shRNA expressing tumor cells reach a volume of ~600 mm^3^ (Fig 4A) and a significant reduction in tumor volume over time (S2A Fig).

To determine the effect of HUNK downregulation on JNK signaling in JIMT-1 cells we next probed for phospho-JNK in control and HUNK shRNA expressing cells and saw a marked reduction in JNK phosphorylation in the HUNK knockdown cells (Fig 4B). We also evaluated EGFR and AKT signaling, as HUNK has been previously implicated in both of these signaling pathways and the activity of those proteins are often deregulated in HER2-inhibitor resistant breast cancers[19, 20]. Our findings show that HUNK knockdown inhibits both EGFR and AKT activity as measured by a reduction in phosphorylation of EGFR (Fig 4C) and AKT (Fig 4D) in the HUNK shRNA expressing JIMT-1 cells compared to control cells.

To evaluate whether co-targeting of JNK and HUNK had an added effect on breast cancer cell death of JIMT-1 cells we next treated control and HUNK knockdown cells with JNK inhibitor and evaluated the cells for Caspase-3 activity. Our findings show that combined inhibition induced cell death to a greater extent than inhibition of JNK alone in control cells or levels of cell death induced by HUNK knockdown alone (Fig 4E). We also evaluated these findings in the BT474-LR (lapatinib resistant) cell line, which we engineered to express control shRNA or shRNA targeted to HUNK (S1B Fig). We confirmed that both the control and HUNK shRNA BT474-LR cells were resistant to lapatinib (S3C Fig) and then we tested them for responsiveness to AKT, JNK, PI3K, MEK, and EGFR inhibitors. Similar to our findings in JIMT-1 cells (Fig 4E) we also saw a marked additive effect of combined HUNK knockdown with the JNK inhibitor (S3D Fig). Furthermore, we saw modest effects on Caspase-3 activation with combined HUNK knockdown and AKT inhibition as well as MEK inhibition (S3D Fig)

**Fig 5:**
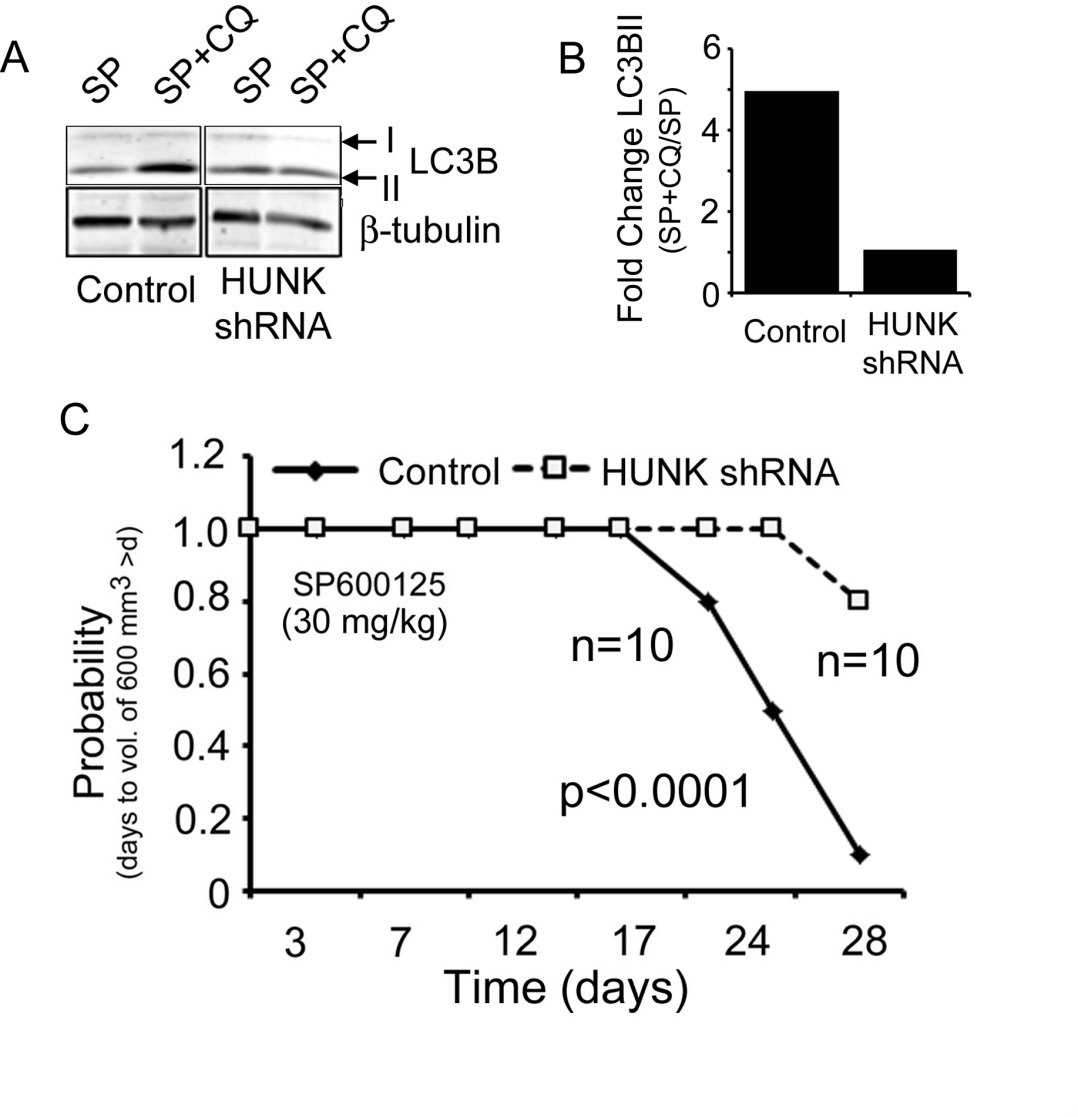
Combined HUNK and JNK targeting induces cell death of JIMT-1 cells and impairs mammary tumor growth. A) Immunoblot analysis of control and HUNK shRNA expressing JIMT-1 cell lysates for LC3B I and II expression after SP600125 (50 μM), or SP600125 and chloroquine (100 ng/ μl) treatment to measure for autophagic flux. B) Quantitation of the fold change in LC3BII levels between the SP600125 and chloroquine treated cells and SP600125 alone treated cells in each cell type, control or HUNK shRNA, to determine the level of autophagic flux in each cell type. C) Time to tumor size of 600 mm^3^ was significantly longer for mice with HUNK shRNA derived tumors treated with SP600125 (30mg/kg) relative to mice with control shRNA derived tumors treated with SP600125 based on Kaplan–meier survival curve estimates; p <0.0001, log-rank test. n = 10, control. n = 10, HUNK shRNA.

We next evaluated whether HUNK knockdown inhibited autophagy and saw a reduction in autophagic flux in HUNK shRNA expressing JIMT-1 cells compared to control cells (Fig 5A), where the fold change in LC3BII accumulation between SP600125 treatment alone and combined SP600125 with chloroquine in the control cells was 5-fold greater than the fold change in accumulation in LC3BII between SP600125 and SP600125 with chloroquine in the Hunk knockdown cells (Fig 5B). These findings suggest that HUNK knockdown impairs autophagy induced by JNK inhibition using autophagic flux as a benchmark measurement as previously described [21].

To evaluate our findings with combined HUNK and JNK inhibition *in vivo* we generated xenograft tumors from the JIMT-1 control and HUNK shRNA expressing cells and treated animals with JNK inhibitor. In this case, we found that our *in vivo* findings were consistent with our *in vitro* findings and treatment of animals harboring HUNK knockdown tumors with SP600125 significantly impaired tumor growth measured by volume (S2B Fig) and delayed median time for the tumors to reach a volume of ~600 mm^3^ by 10.3 days beyond that of SP699125 treatment in animals injected with control cells (Fig 5C). Taken together, these findings implicate HUNK targeting as an effective combination that can be paired with JNK inhibition in HER2-inhibitor resistant breast cancers.

## Discussion

c-Jun N-terminal kinase (JNK), is a serine-threonine kinase that belongs to the mitogen-activated protein kinase (MAPK) family of protein kinases. It was originally shown to regulate c-Jun, which is a member of the activating protein 1 (AP-1) family of transcription factors. A variety of stress signals including inflammatory signals, changes in reactive oxygen species, ultraviolet radiation, and protein synthesis inhibitors activate JNK leading to regulation of cell death, survival, proliferation, and inflammatory response. Many lines of evidence point to JNK as having a role in cellular transformation and human cancer. Early findings indicate that the transforming actions of several oncogenes including Ras and Bcr-Abl depend on JNK and its downstream target c-Jun [22–24]. Furthermore, hyperactivation of JNK has been reported in multiple cancer cell lines and tissue samples, suggesting a pro-tumorigenic role for this kinase. JNK hyperactivation and/or overexpression is observed in hepatocellular carcinoma, squamous cell carcinoma, and glioblastoma [25]

Interestingly, JNK has also been described as having a tumor suppressive role, in part because in some mammary tumor models, the inhibition of the JNK pathway has been described to promote tumorigenesis. Using a genetically engineered mouse model heterozygous for p53 (p53+/-) crossed with JNK knockout (JNK1-/- or JNK2-/-) animals, Cellurale et al demonstrated that the resulting p53+/;JNK1-/- and p53+/-;JNK2-/- mice had reduced tumor-free survival compared to p53+/- mice that express wildtype JNK [26]. Evidence from the MMTV- polyoma middle T (PymT) transgenic model also showed a tumor suppressive function for JNK as MMTV-PymT; JNK2-/- displayed shortened tumor latency and increased tumor multiplicity when compared to MMTV-PymT control mice [27]. Additional evidence shows that inactivation of the upstream activator of JNK, MKK7, in MMTV-neu mice caused earlier onset of tumors and reduced overall survival [28]. However, contradictory evidence from a publication by Han and Crowe shows that in the MMTV-neu background, JNK1 expression is high and inhibiting JNK1 increased tumor latency and decreased tumor growth [29], indicating that pro-tumorigenic functions for JNK could also exist in *neu*-driven mammary cancers. In support of this observation, studies in the 4T1 mammary tumor cell model showed that downregulation of JNK2 with shRNA inhibited tumor growth as well as metastasis to the lung of xenograft mammary tumors [30]. Similarly, inhibition of JNK2 using a JNK2 selective inhibitor in MMTV-PymT-derived mammary tumor cells reduced cell migration [31]. Our current findings are in agreement with the observation that JNK is pro-tumorigenic since we demonstrate that inhibition of JNK with SP600126 in HER2-positive cells that are resistant to trastuzumab and lapatinib, impairs tumor growth. Coincident with this finding we see upregulation of JNK protein and phosphorylation in JIMT-1 resistant cells when compared to BT474, HER2 inhibitor sensitive cells suggesting that JNK expression is increased in the JIMT-1 resistant cell line, perhaps as a mode of survival.

One potential reason for the discrepancy in JNK’s role in tumorigenesis could be due to its roles in regulating apoptosis. JNK has been shown to perform opposing roles in apoptosis as it has both pro- and anti-apoptotic functions [32]. A possible explanation for why JNK can function as an anti-apoptotic protein in some tumors could be related to p53 status, where JNK inhibition prohibits the growth of p53-deficient or -dysfunctional tumors but not those containing wildtype p53 [33]. Certainly evidence suggests that coincident loss of p53 function or p53 mutation in HER2/neu-positive breast cancers is likely frequent and is associated with poor survival [34–36], which could explain our findings that JNK inhibition by SP600125 treatment induces Caspase-3-dependent cell death and impairs tumor growth in the JIMT-1 model as these cells are mutant for *TP53* [37]. Overall our findings indicate that JNK acts as a pro-survival molecule in the two resistance models we use; JIMT-1 and BT474-LR. This finding is at odds with studies suggesting that JNK is pro-apoptotic in certain mammary tumor models [26–28]. However, studies evaluating pro-death functions of JNK do not necessarily take into account other modes of cell death, such as autophagy, which are regulated by JNK.

Studies also indicate that JNK regulates cell death via autophagy. Previous findings from Cufi et al show that autophagy is upregulated in JIMT-1 cells [17]. Our finding that JNK protein and phosphorylation are higher in JIMT-1 cells compared to BT474 cells is correlative with the finding that autophagy is also upregulated. However, under current paradigms JNK upregulates autophagy to promote autophagy-mediated cell death [38–40]. Our findings would suggest that, similar to apoptosis, JNK could have a dual role in autophagy by also stimulating autophagy-mediated cell survival. Interestingly, in our study, treatment of JIMT-1 cells with the JNK inhibitor SP600125 further upregulated autophagy. Furthermore, we were surprised to find that inhibiting autophagy with chloroquine did not add any benefit to SP600125 treatment in *in vivo* mammary tumor growth analysis, but, this finding perhaps speaks to the need to identify more specific mechanistic interactions between JNK and autophagy regulatory proteins in order to effectively modulate JNK activity and autophagy proteins in human breast cancer.

Similar to chloroquine, when we treated JIMT-1 cells with the combination of SP600125 and lapatinib, we did not see an effect on mammary tumor growth *in vivo*. This observation is perhaps not surprising if the added stress of lapatinib upregulates additional nodes of stress induced survival that circumvent JNK inhibition, including autophagy. Consistent with this supposition, we saw an increase in JNK phosphorylation in response to lapatinib treatment and despite the loss of this effect when both the JNK and HER2 inhibitor were used, there was no change in tumorigenesis. One might conclude that since both JNK and lapatinib upregulate autophagy that this response could outweigh any benefit of using both inhibitors.

In line with this idea, we were able to demonstrate that combined targeting of JNK with the protein kinase HUNK significantly impaired tumorigenesis using the JIMT-1 model. Previously it was shown that HUNK regulates autophagy in BT474, HER2+ cells that are sensitive to HER2-inhibition, as well as in JIMT-1 cells [16]. While a defined mechanism for how HUNK regulates autophagy has not yet been delineated, our findings could suggest that modulation of autophagy at different stages of the autophagy process may result in distinct outcomes. Specifically, while HUNK inhibition was able to work in concert with JNK inhibition, combined chloroquine and SP600125 treatment did not. This difference in response could be explained by the mechanism of action of chloroquine as it is a more general autophagy inhibitor that impacts the entire lysosomal system, and therefore may affect other lysosomal-mediated processes. The difference observed between chloroquine treatment and HUNK knockdown could be dependent on which stage of autophagy is being inhibited; early autophagy for HUNK versus late autophagy for chloroquine. However, additional experiments to evaluate exactly what point in autophagy HUNK is acting remain to be explored.

In terms of therapeutic interventions for HER2-inhibitor resistant breast cancers, we have provided evidence that targeting JNK with the pan-JNK inhibitor SP600125 was effective in delaying tumor growth in the xenograft JIMT-1 mammary tumor model system. Moreover, identifying signaling molecules that can be effectively co-targeted such as HUNK kinase, which we demonstrate here can have an additive effect on impairing tumor growth when paired with SP600125, could be an effective strategy in treating HER2+ breast cancers that have become resistant to HER2 inhibition. Future studies with an improved, clinically relevant JNK inhibitor as well as a chemical inhibitor for HUNK are required for further assessment of treatment potential.

## Supporting Information

S1 Fig HUNK knockdown levels in JIMT-1 and BT474-LR cells. A) *HUNK* mRNA levels in JIMT-1 cells engineered to express a control shRNA or shRNA targeted to HUNK. B) *HUNK* mRNA levels in BT474-LR cells engineered to express a control shRNA or shRNA targeted to HUNK.
(TIF)

S2 Fig Tumor volume analysis of JIMT-1 control and HUNK shRNA mammary tumor xenografts. A) Tumor volume curve comparing immunocompromised mice injected with JIMT-1 tumor cells expressing control shRNA or shRNA targeted to HUNK. B) Tumor volume comparison of immunocompromised mice injected with JIMT-1 tumor cells expressing control shRNA or shRNA targeted to HUNK at day 14. Both cohorts of animals were treated with 30 mg/kg SP600125.
(TIF)

S3 Fig Evaluation of BT474-LR cells. A) BT474-LR cells were compared to BT474 parental cells for sensitivity to lapatinib at 1 μM concentration. Cell viability was measured by crystal violet 24 hrs after lapatinib treatment. OD 540 was quantitated to demonstrate amount of cells present and able to take up dye. p-values were determined by student’s T-test. B) Lapatinib resistant BT474 cells (BT474-LR) treated with inhibitors targeting AKT, PLC, JNK, and PI3K for 24 hrs and evaluated for Caspase-3 activity as a measure of apoptosis. p-values were determined by student’s T-test. C) Control and HUNK shRNA expressing BT474-LR cells were treated with 1 μM lapatinib for 24 hrs and evaluated for Caspase-3 activity as a measure of apoptosis. p-values were determined by student’s T-test. D) Control and HUNK shRNA expressing BT474-LR cells were treated with inhibitors targeting AKT, PLC, JNK, PI3K, MEK, and EGFR for 24 hrs and evaluated for Caspase-3 activity as a measure of apoptosis. p-values were determined by student’s T-test.
(TIF)
