# Supplementary figures and images for: Co-Targeting of JNK and HUNK in Resistant HER2-Positive Breast Cancer
Source: PLoS One. 2016 Apr 5;11(4):e0153025. doi: 10.1371/journal.pone.0153025 (PMC4821489; doi:10.1371/journal.pone.0153025)

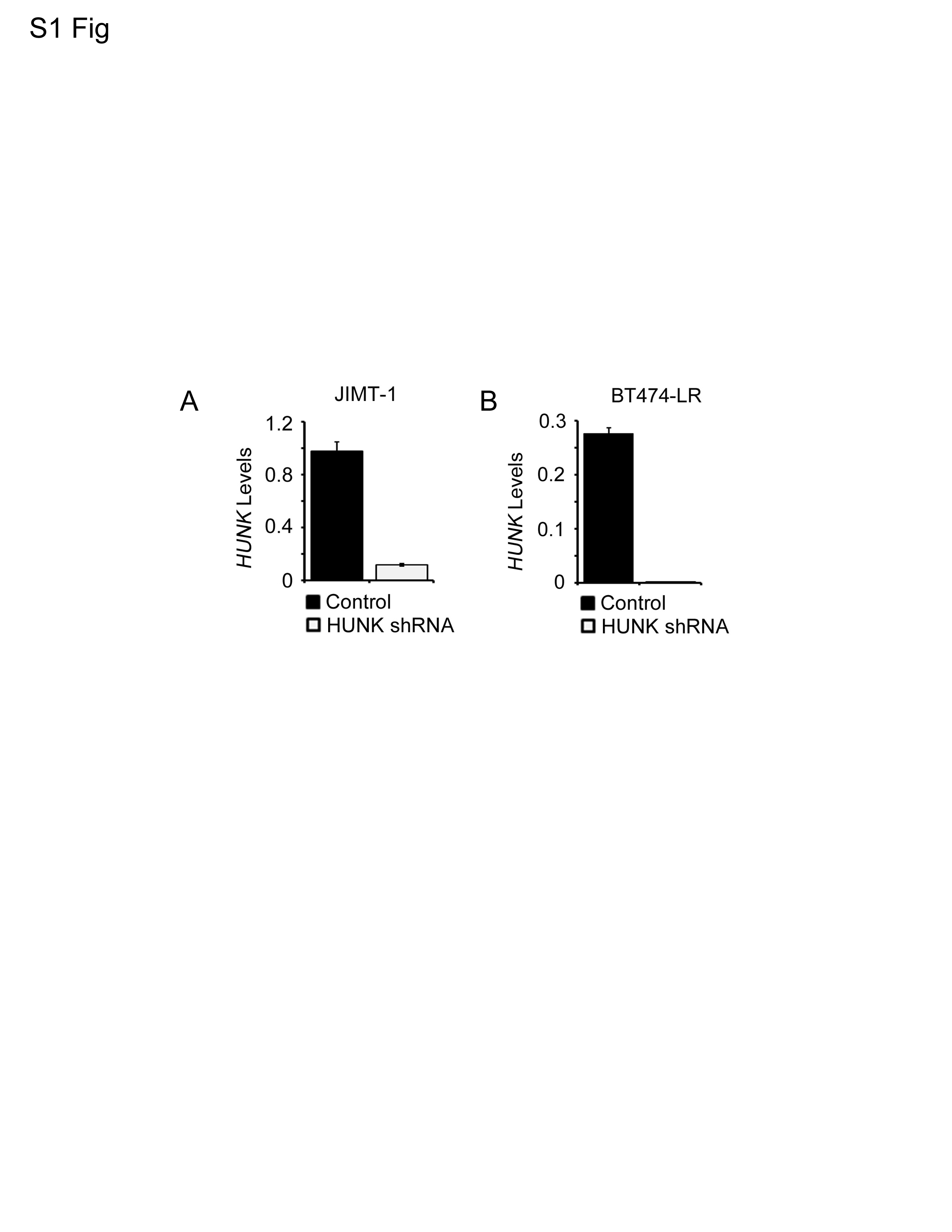

Supplement: S1 Fig — A) HUNK mRNA levels in JIMT-1 cells engineered to express a control shRNA or shRNA targeted to HUNK. B) HUNK mRNA levels in BT474-LR cells engineered to express a control shRNA or shRNA targeted to HUNK. (TIF) [file pone.0153025.s001.tif]

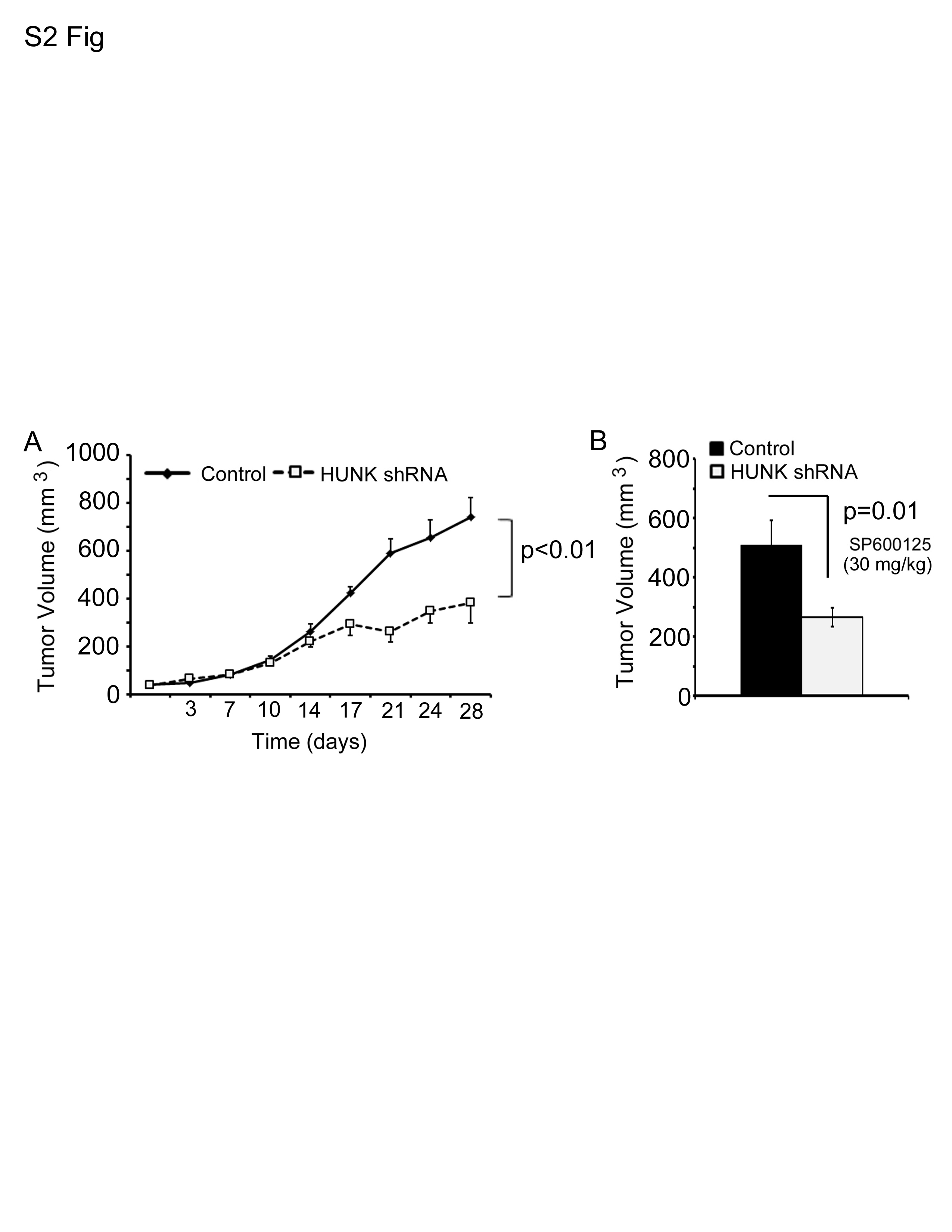

Supplement: S2 Fig — A) Tumor volume curve comparing immunocompromised mice injected with JIMT-1 tumor cells expressing control shRNA or shRNA targeted to HUNK. B) Tumor volume comparison of immunocompromised mice injected with JIMT-1 tumor cells expressing control shRNA or shRNA targeted to HUNK at day 14. Both cohorts of animals were treated with 30 mg/kg SP600125. (TIF) [file pone.0153025.s002.tif]

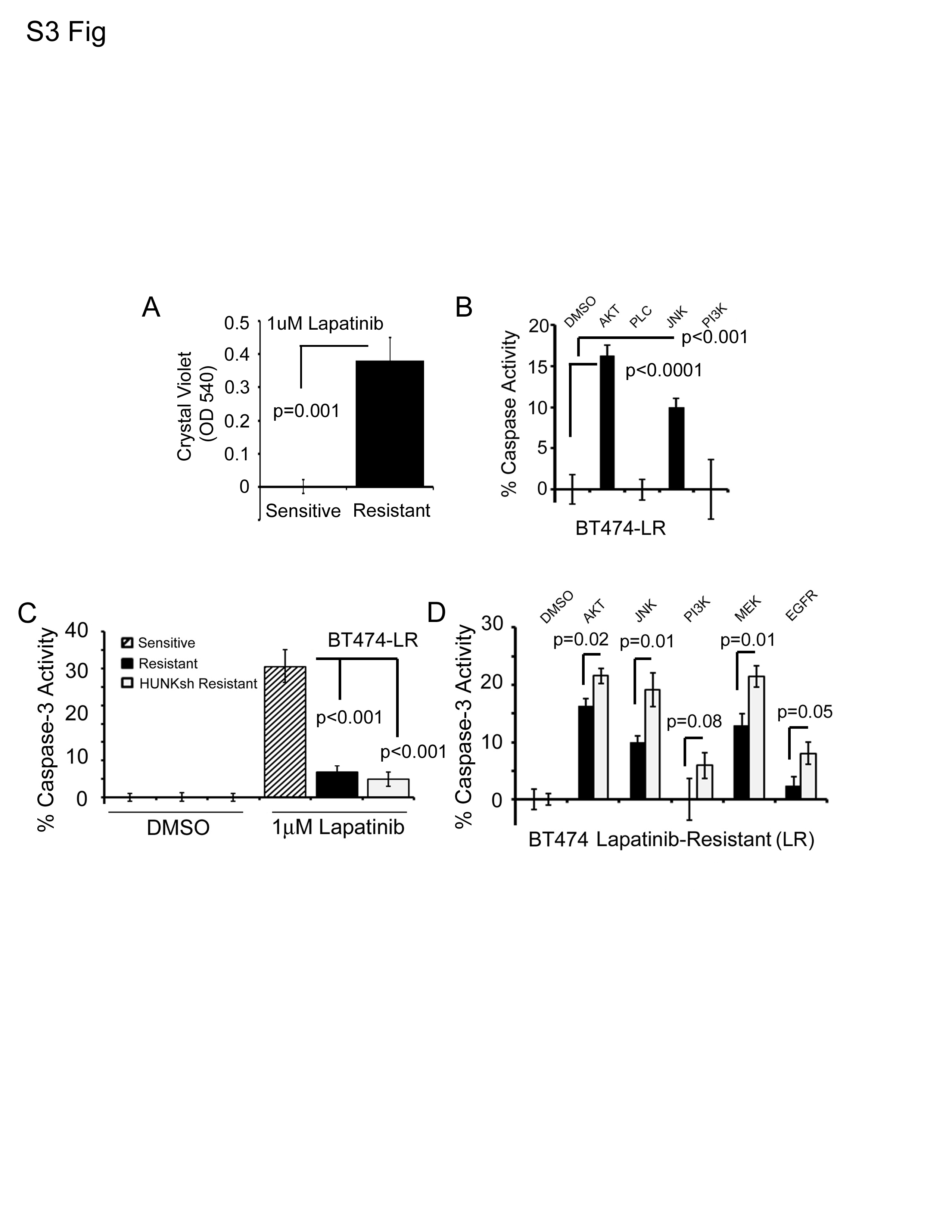

Supplement: S3 Fig — A) BT474-LR cells were compared to BT474 parental cells for sensitivity to lapatinib at 1 μM concentration. Cell viability was measured by crystal violet 24 hrs after lapatinib treatment. OD 540 was quantitated to demonstrate amount of cells present and able to take up dye. p-values were determined by student’s T-test. B) Lapatinib resistant BT474 cells (BT474-LR) treated with inhibitors targeting AKT, PLC, JNK, and PI3K for 24 hrs and evaluated for Caspase-3 activity as a measure of apoptosis. p-values were determined by student’s T-test. C) Control and HUNK shRNA expressing BT474-LR cells were treated with 1 μM lapatinib for 24 hrs and evaluated for Caspase-3 activity as a measure of apoptosis. p-values were determined by student’s T-test. D) Control and HUNK shRNA expressing BT474-LR cells were treated with inhibitors targeting AKT, PLC, JNK, PI3K, MEK, and EGFR for 24 hrs and evaluated for Caspase-3 activity as a measure of apoptosis. p-values were determined by student’s T-test. (TIF) [file pone.0153025.s003.tif]
